# Association between informal employment and depressive symptoms in 11 cities in Latin America

**DOI:** 10.1016/j.ssmph.2022.101101

**Published:** 2022-04-20

**Authors:** Tran B. Huynh, Vanessa M. Oddo, Bricia Trejo, Kari Moore, D. Alex Quistberg, Jannie J. Kim, Francisco Diez-Canseco, Alejandra Vives

**Affiliations:** a Department of Environmental and Occupational Health, Dornsife School of Public Health, Drexel University, Philadelphia, PA, USA; b Department of Kinesiology and Nutrition, University of Illinois Chicago, Chicago, IL, USA; c Urban Health Collaborative, Dornsife School of Public Health, Drexel University, Philadelphia, PA, USA; d CRONICAS Centre of Excellence in Chronic Diseases, Universidad Peruana Cayetano Heredia, Lima, Peru; e Department of Public Health, School of Medicine / CEDEUS, Pontificia Universidad Católica de Chile, Santiago, Chile; f Department of Epidemiology and Biostatistics, Dornsife School of Public Health, Drexel University, Philadelphia, PA, USA

**Keywords:** Informal employment, Depression, Mental health, Latin American and the Caribbean

## Abstract

**Background:**

Mental health is an important contributor to the global burden of disease, and depression is the most prevalent mental disorder in Latin America and the Caribbean (LAC). Informal jobs, often characterized by precarious working conditions, low wages, and limited employment benefits, are also highly prevalent in LAC and may be associated with poorer mental health. Our study tests the association between informal employment and major depressive symptoms in LAC cities.

**Methods:**

We used individual-level data collected by the Development Bank of Latin America via their “Encuesta CAF” (ECAF) 2016, a cross-sectional household survey of 11 LAC cities (N = 5430). Depressive symptoms were measured using the 10-item Center for Epidemiologic Studies Short Depression Scale with possible total score ranging from 0 to 30. Scores were dichotomized, with a score $\underset{‾}{\text{>}}$ 16 indicating the presence of major depressive symptoms. Informal employment was defined based on self-reported lack of contribution to the social security system. We used generalized estimating equation (GEE) log-binomial models to estimate the association between informal employment and depressive symptoms overall and by gender. Models were adjusted for age, education, and household characteristics.

**Results:**

Overall, individuals employed in informal jobs had a 27% higher prevalence of major depressive symptoms (Prevalence Ratio [PR]: 1.27; 95% Confidence Interval [CI]: 1.00, 1.62) compared to those in formal jobs. The prevalence of depressive symptoms among individuals with informal jobs was higher compared to those with formal jobs in both women (PR: 1.36, 95% CI: 1.06, 1.74) and men (PR: 1.22; 95% CI: 0.90, 1.65).

**Conclusions:**

Informal employment in LAC was associated with a higher prevalence of major depressive symptoms. It is important to develop policies aiming at reducing informal jobs and increasing universal social protection for informal workers.

## Highlights

• Informal employment is associated with a higher prevalence of depressive symptoms in Latin America.
• Associations for both men and women were similar in direction and magnitude but only among women was the association statistically significant.

## 1 Introduction

Mental health is increasingly recognized an important contributor to the global burden of disease and is a key component of individual health and well-being that has been included in the Sustainable Development Goals (Votruba et al., 2016). In Latin America and the Caribbean (LAC), approximately 20% of the disease burden, measured by disability-free life years lost, can be attributed to mental and substance use disorders (Kohn et al., 2018). Estimates indicate that the 12-month prevalence of mental disorders in LAC is approximately 15% (Kohn et al., 2018) and that depression is the most prevalent mental disorder in the region (Pan American Health Organization (PAHO), 2012). Moreover, the prevalence of mental health disorders in large urban areas may be even higher; for example, in São Paulo, an area with more than 21 million inhabitants, the 12-month prevalence of any mental health disorder is an estimated 30% (Andrade et al., 2012). Despite this, approximately 65% of people who need care for depression in LAC do not receive it (PAHO, 2012).

Increasingly, informal employment is recognized as a social determinant of health (Lahiri et al., 2006; Saunders, Barr, McHale, & Hamelmann, 2017; Vélez Álvarez et al., 2013) and prior studies have indicated that those who are informally employed or in precarious work may have poorer mental health outcomes (Vives et al., 2011, 2013; Muntaner et al., 2020). In LAC, approximately 50% of the population are engaged in informal employment (Bonnet et al., 2019), which is commonly defined as jobs that are not subject to national law regulations, taxation, and social protections (Bonnet et al., 2019). Informal working conditions often reflect this lack of regulation, as informal employment tends to include jobs that are insecure, low-wage and lack benefits (e.g., paid sick leave, severance pay, paid annual leave).

Despite the high prevalence of both informal employment and mental health disorders, relatively few studies have investigated the extent to which informal employment is associated with mental health in LAC and those prior studies report mixed results. While some studies suggest that informal employment is associated with poorer mental health (da Silva et al., 2006; Santana, 1997; Rodriguez-Loureiro et al., 2020), one study reports variation in the association depending on the indicator of informal employment being used (e.g., social security coverage, or types of contract) and/or the mental health outcome being measured (López-Ruiz et al., 2015). Other evidence suggests heterogeneity in the association by gender (Lopez-Ruiz et al., 2017, Ludermir & Lewis, 2005, Ruiz et al., 2017). In Central America, Lopez-Ruiz and colleagues reported that among women in informal employment, there was positive association between long work hours, part-time work and poorer mental health, but no association among men (Lopez-Ruiz et al., 2017). Likewise, Ludermir and Lewis (2005) in Brazil reported an association between informal work and common mental disorders among women but not men (Ludermir & Lewis, 2005). On the contrary, in Chile, the association between informal employment and mental health was observed among men but not women (Ruiz et al., 2017).

We build upon prior literature by utilizing a unique multi-city data source to test the association between informal employment and major depressive symptoms in urban areas in LAC. We hypothesized that informal workers have a higher prevalence of depressive symptoms compared to their formally employed counterparts. Understanding the extent to which informal employment is related to depressive symptoms is critical for the development of public policies and intervention strategies aimed at improving working conditions and mental health in the region.

## 2 Methods

### 2.1 Data source

The data in this study were compiled by the SALURBAL, Salud Urban en America Latina (Urban Health in Latin America), project based at Drexel University in Philadelphia, Pennsylvania, USA and 14 partner institutions across Latin American countries (Diez Roux et al., 2019). We utilized data collected by the Development Bank of Latin America (*Corporación Andina de Fomento (CAF)*, n.d.) via their “Encuesta CAF” (ECAF) 2016 cross-sectional household survey (CAF, 2016). Data collection occurred between November 2016 and January 2017 in 11 major cities in Latin America: Bogotá (Colombia), Buenos Aires (Argentina), Caracas (Venezuela), Fortaleza (Brazil), La Paz (Bolivia), Lima (Peru), México DF (Mexico), Montevideo (Uruguay), Panamá (Panama), Quito (Ecuador), and São Paulo (Brazil) (CAF, 2016).

The sampling procedure is described in detail elsewhere (CAF, 2016). Briefly, the sample was stratified by city, based in the main city areas and, in some cases, on the socioeconomic status. The sampling was designed to be representative of the urban population between 20 and 60 years old in each city, and quotas were defined by gender and age (i.e., four age groups). In each household, only one person (aged 20–60 years old) was interviewed.

### 2.2 Outcome and exposure

*Outcome:* The outcome variable was a dichotomous indicator of self-reported major depressive symptoms, which were measured by the 10-item Center for Epidemiologic Studies Short Depression Scale (CES-D-10), a screening tool demonstrated to have high reliability and validity for identifying individuals with major depressive symptoms in general populations (Björgvinsson et al., 2013,Andresen et al., 2013; Radloff, 1977). The Spanish version has been validated in Hispanic/Latino adults in the US (González et al., 2017), Puerto Rican adults in Connecticut (Robison et al., 2002), adults in the Andean region of Bolivia (Schantz et al., 2017), and indigenous population of Mexico (Franco-Díaz et al., 2018). The mental health section of the CAF survey consisted of 10 questions relating to how often respondents experienced symptoms of depression during the past week on a four-point Likert scale (“less than 1 day”, “1–2 days”, “3–4 days”, “5–7 days”). The responses were scored according to the CES-D-10 standard procedure where the two positive questions were reversed coded and a total score for each participant was summed over the 10 items with possible total score of 0–30. A higher score reflects higher level of depression. The suggested cutoff point to indicate significant depressive symptoms varies in the literature ranging from 10 to 16 (Andresen et al., 2013; Björgvinsson et al., 2013; Cheng & Chan, 2005 Weiss et al., 2015; Zhang et al., 2012). We chose the cutoff point of 16 for optimal sensitivity and specificity. Thus, respondents’ scores were dichotomized with a score $\underset{‾}{\text{>}}$ 16 indicating of the presence of major depressive symptoms (Weiss et al., 2015).

*Exposure.* Our primary exposure variable was a dichotomous indicator of informal employment. Consistent with prior literature, informal employment was defined based on individuals' contribution to social security systems (e.g., Bonnet et al., 2019; López-Ruiz et al., 2015; Rodriguez-Loureiro et al., 2020). Among those who reported being employed, respondents were asked, “Does your employer or you make contributions to your retirement/pension fund/social security?” whereby those responding “yes” were categorized as formally employed and those responding “no” were considered informally employed.

### 2.3 Confounders and effect modifiers

Based on prior literature, gender was considered as an effect modifier a priori for work-related risk of mental health. Women in LAC are generally paid less than men with the same qualifications (ILO, 2019) and are more likely to be employed in lower-quality jobs (e.g., part-time, informal, temporary), with less protection and job security, while also facing a greater burden of family and work responsibilities (ILO, 2019). Moreover, the work environment for men and women are systematically different across and within occupations in terms of the labor distribution and job tasks. Additionally, inequality in gender and socioeconomic status could result in the uneven distribution of social support and subsequently health (Bolibar et al., 2021).

At the same time, depression is more prevalent in adult women versus men globally and also in LAC (Brody, 2018; Lopez-Ruiz et al., 2017; Moreno-Agostino et al., 2021; Piccinelli & Wilkinson, 2000; Rodriguez-Loureiro et al., 2020; Utzet et al., 2021) and social and economic inequalities have been implicated as major causes for the gender disparity in depression (Eugenia Alvarado et al., 2007; Walters et al., 2002).

We then identified confounding factors a priori using a directed acyclic graph, which is a causal diagram used to identify factors that affect both the exposure (informal employment) and the outcome (depression), based on theorized relationships and relationships documented in the literature (Greenland et al., 1999). Informed by existing literature, plausible confounders included age (categorical), education (categorical), household size (continuous), relationship status (binary), and having children under 5 years old in the household (binary).

Notably, we hypothesized that household income was a mediator of the informal employment-depression association (i.e., on the causal pathway); formal versus informal employment would likely result in increased individual-level income, thus, influence depression risk. Similarly, employment-related characteristics such as occupation categories, hours worked per week and work location could be potential mediators, i.e., on the causal pathway of the informal employment-depression association. We present information on these characteristics descriptively, but these potential mediators were not included in our multivariable regression models.

### 2.4 Analytic sample

Persons eligible for the interviews were the heads of the household or adults aged 20–60 years (N = 12,905). We excluded individuals who were missing employment status (N = 32), who responded that they were unemployed (N = 1239), those who self-reported that they were currently “inactive” or have not looked for a job during the last 4 weeks (N = 3582), and those who never responded (N = 109). Of the 7979 who reported being employed, we further excluded those with no information on type of employment (N = 221) and those missing >5 questions on the CESD-10 (N = 1720). For individuals with ≤ 5 missing CESD-10 questions (n = 858), we imputed missing values using mean imputation (Bono et al., 2007; Rush Alzheimer's Disease Center (RADC) Research Resource Sharing Hub, n.d.). We also excluded those with missing demographic covariates (age, gender, education) and household composition covariates (relationship status, household size, children in the household under 5 years old) (N = 80), and those missing data on other employment characteristics (e.g., occupation) (N = 528). The final analytic sample consisted of 5430 individuals. The characteristics of the analytic sample were similar to the 7979 eligible with respect to education, employment type, age and gender (Supplemental Table 1).

### 2.5 Statistical analysis

We present the sample sizes, frequencies (N, %), and means (standard deviation) for descriptive statistics and test for group differences between formal versus informal employment. We used t-tests for continuous variables (means) and chi-squared for categorical variables (percents).

In our primary specification, we estimated log-binomial models, employing a generalized estimating equation (GEE) approach, with city-clustered standard errors (McNutt et al., 2003). We estimated the association both overall and gender-stratified. First, in a minimally adjusted model, we tested the association between informal employment and depression controlling for age and education (and gender for the overall models). Second, our model adjusted for the aforementioned demographic variables, as well as household-level covariates, including relationship status, household size and having a child under the age of 5 in the household. As a sensitivity analysis, we estimated the aforementioned models with an un-imputed sample (i.e., complete cases) (N = 4572) and when excluding education as a covariate.

Alpha was set to 0.05. All analyses were run in R Version April 1, 1103. This study was approved by the Drexel University Institutional Review Board with ID #1612005035 and by appropriate site-specific IRBs across academic institutions included in the SALURBAL project.

## 3 Results

### 3.1 Descriptive results

Table 1 details selected characteristics of the sample stratified by formal/informal employment, both overall and further stratified by gender. Overall, informally employed individuals had a higher prevalence of major depressive symptoms (14.4%), compared to formally employed (10.9%). This trend was also observed across gender; 17.0% of informally employed women had major depressive symptoms compared to 12.8% of formally employed women. Likewise, the prevalence of major depressive symptoms was 12.1% and 9.5% among informally versus formally employed men, respectively.

**Table 1:** Selected sample characteristics of formal and informal employment, overall and stratified by gender.

	N (%) or Mean (Standard Deviation)^a^^,^^b^								
	Overall			Women			Men		
	Formal	Informal	*p*	Formal	Informal	*p*	Formal	Informal	*p*
	(N = 2588)	(N = 2842)		(N = 1056)	(N = 1294)		(N = 1532)	(N = 1548)	
**Depressive Symptoms**^c^			<0.01			<0.01			<0.01
No	2307 (89.1%)	2434 (85.6%)		921 (87.2%)	1074 (83.0%)		1386 (90.5%)	1360 (87.9%)	
Yes	281 (10.9%)	408 (14.4%)		135 (12.8%)	220 (17.0%)		146 (9.5%)	188 (12.1%)	
**Mean CES-D-10 Score**^d^	7.7 (5.9)	8.8 (5.9)	<0.01	8.3 (6.0)	9.3 (6.1)	<0.01	7.3 (5.8)	8.3 (5.6)	<0.01
**Gender**									
Women	1056 (40.8%)	1294 (45.5%)	<0.01						
Men	1532 (59.1%)	1548 (54.5%)							
**Age**			0.01			0.03			0.07
20-29	723 (27.9%)	782 (27.5%)		285 (27.0%)	329 (25.4%)		438 (28.6%)	453 (29.3%)	
30-39	826 (31.9%)	828 (29.1%)		345 (32.7%)	369 (28.5%)		481 (31.4%)	459 (29.7%)	
40-49	643 (24.8%)	703 (24.7%)		270 (25.6%)	359 (27.7%)		373 (24.3%)	344 (22.2%)	
50-60	396 (15.3%)	529 (18.6%)		156 (14.8%)	237 (18.3%)		240 (15.7%)	292 (18.9%)	
**Education**			<0.01			<0.01			<0.01
Less than Primary	67 (2.6%)	255 (9.0%)		26 (2.5%)	125 (9.7%)		41 (2.7%)	130 (8.4%)	
Primary Complete	609 (23.5%)	1084 (38.1%)		219 (20.7%)	476 (36.8%)		390 (25.5%)	608 (39.3%)	
Secondary Complete	982 (37.9%)	1066 (37.5%)		380 (36.0%)	493 (38.1%)		602 (39.3%)	573 (37.0%)	
> Secondary	930 (35.9%)	437 (15.4%)		431 (40.8%)	200 (15.5%)		499 (32.6%)	237 (15.3%)	
**Mean Household Size**	3.9 (1.6)	4.2 (1.9)	<0.01	3.8 (1.5)	4.3 (1.8)	<0.01	3.9 (1.7)	4.2 (2.0)	<0.01
**Relationship Status**			0.03			0.77			<0.01
In a relationship	1854 (71.6%)	1959 (68.9%)		699 (66.2%)	865 (66.8%)		1155 (75.4%)	1094 (70.7%)	
Not in a relationship	734 (28.4%)	883 (31.1%)		357 (33.8%)	429 (33.2%)		377 (24.6%)	454 (29.3%)	
**Children** < **5 yrs in household**			<0.01			<0.01			0.04
No	1786 (69.0%)	1796 (63.2%)		739 (70.0%)	792 (61.2%)		1047 (68.3%)	1004 (64.9%)	
Yes	802 (31.0%)	1046 (36.8%)		317 (30.0%)	502 (38.8%)		485 (31.7%)	544 (35.1%)	
**City**			<0.01			<0.01			<0.01
Bogota	398 (15.4%)	321 (11.3%)		178 (16.9%)	202 (15.6%)		220 (14.4%)	119 (7.7%)	
Buenos Aires	294 (11.4%)	420 (14.8%)		119 (11.3%)	172 (13.3%)		175 (11.4%)	248 (16.0%)	
Caracas	288 (11.1%)	314 (11.0%)		130 (12.3%)	118 (9.1%)		158 (10.3%)	196 (12.7%)	
Fortaleza	166 (6.4%)	280 (9.9%)		69 (6.5%)	139 (10.7%)		97.0 (6.3%)	141 (9.1%)	
La Paz	133 (5.1%)	368 (12.9%)		39 (3.7%)	169 (13.1%)		94.0 (6.1%)	199 (12.9%)	
Lima	169 (6.5%)	305 (10.7%)		51(4.8%)	119 (9.2%)		118 (7.7%)	186 (12.0%)	
Mexico	150 (5.8%)	149 (5.2%)		43 (4.1%)	67 (5.2%)		107 (7.0%)	82 (5.3%)	
Montevideo	399 (15.4%)	116 (4.1%)		196 (18.6%)	56 (4.3%)		203 (13.3%)	60 (3.9%)	
Panama	102 (3.9%)	88 (3.1%)		41 (3.9%)	32 (2.5%)		61 (4.0%)	56 (3.6%)	
Quito	162 (6.3%)	266 (9.4%)		59 (5.6%)	117 (9.0%)		103 (6.7%)	149 (9.6%)	
São Paulo	327 (12.6%)	215 (7.6%)		131 (12.4%)	103 (8.0%)		196 (12.8%)	112 (7.2%)	

CES-D-10 = 10-item Center for Epidemiologic Studies Short Depression Scale.

a Informal employment was defined as no contribution to social security systems. *p* values represent any group differences using t-tests (for mean [SD]), and chi-squared (for percents).

b Missing values: overall (formal n = 11, informal n = 38), Females (formal n = 3, informal n = 20), Males (formal n = 8, informal n = 18).

c Defined as CES-D-10 score $\underset{‾}{\text{>}}$ 16.

d Possible range: 0 to 30.

Overall, about one-quarter of formally and informally employed individuals were aged 20–29 years and about 30% were aged 30–39 years (Table 1). This age distribution was similar by gender. Most formally (71.6%) and informally (68.9%) individuals were in a relationship. More than 35% of formally employed individuals had achieved greater than a secondary level of education, compared to 15.4% of informally employed individuals. This trend was also generally similar by gender. On average, household size was smaller among those who were formally employed (mean = 3.9; standard deviation [SD] = 1.6) compared to those engaged in informal employment (mean = 4.2; SD = 1.9). Among formally employed individuals, 31.0% had children under the age of 5 in the household, compared to 36.8% of informally employed individuals.

Supplemental Table 2 details the sample characteristics only stratified by gender. A majority (55.1%) of women were employed in the informal sector, whereas men were equally (∼50%) distributed in both informal and formal sectors. Women had higher mean depressive score (8.9; SD = 6.1) than men (7.8; SD = 5.7).

Table 2 details selected employment characteristics of the sample stratified by formal/informal employment, both overall and further stratified by gender. A large majority of formally employed individuals worked in a permanent place outside the home (83.3%) whereas work location was mixed among the informally employed (e.g., 24.6% working at home, 26.0% no fixed work location). About a quarter (24.6%) of formally employed individuals worked in qualified non-manual jobs (i.e., more skilled) (see Supplemental Table 3 for job categorizations). Smaller proportions of informally employed individuals worked in qualified non-manual jobs (6.8%). About one-third (36.2%) of informally employed individuals worked in non-qualified non-manual (i.e., less skilled) and non-qualified manual (39.7%) jobs (i.e., the least skilled). More informally employed individuals (74.4%) reported to be self-employed; whereas 61.0% formally employed reported to have an employer. Approximately 11% of the formally and informally employed individuals had a second job.

**Table 2:** Employment characteristics for formal and informal employment, overall and stratified by gender.

	N (%) or Mean (Standard Deviation)^a^								
	Overall			Women			Men		
	Formal	Informal	*p*	Formal	Informal	*p*	Formal	Informal	*p*
	(N = 2588)	(N = 2842)		(N = 1056)	(N = 1294)		(N = 1532)	(N = 1548)	
**Mean Hours/Week**	45.0 (14.3)	44.6 (21.3)	0.47	41.9 (14.3)	41.6 (23.4)	0.68	47.1 (13.9)	47.2 (19.0)	0.90
**Work Location**			<0.01			<0.01			<0.01
At home	150 (5.8%)	698 (24.6%)		74.0 (7.0%)	440 (34.0%)		76.0 (5.0%)	258 (16.7%)	
Fixed kiosk in public street	56 (2.2%)	209 (7.4%)		19.0 (1.8%)	85.0 (6.6%)		37.0 (2.4%)	124 (8.0%)	
No fixed place	226 (8.7%)	739 (26.0%)		56.0 (5.3%)	256 (19.8%)		170 (11.1%)	483 (31.2%)	
Permanent place outside home	2156 (83.3%)	1196 (42.1%)		907 (85.9%)	513 (39.6%)		1249 (81.5%)	683 (44.1%)	
**Mean Years in Job**^b^	7.3 (7.9)	7.4 (8.5)	0.88	6.7 (7.5)	5.9 (7.5)	0.02	7.8 (8.2)	8.6 (9.0)	0.01
**Occupation Type**^c^			<0.01			<0.01			<0.01
Qualified nonmanual	637 (24.6%)	193 (6.8%)		309 (29.3%)	82.0 (6.3%)		328 (21.4%)	111 (7.2%)	
Nonqualified nonmanual	1070 (41.3%)	1029 (36.2%)		492 (46.6%)	600 (46.4%)		578 (37.7%)	429 (27.7%)	
Qualified manual	325 (12.6%)	491 (17.3%)		17.0 (1.6%)	38.0 (2.9%)		308 (20.1%)	453 (29.3%)	
Nonqualified manual	556 (21.5%)	1129 (39.7%)		238 (22.5%)	574 (44.4%)		318 (20.8%)	555 (35.9%)	
**Employment Status**			<0.01			<0.01			<0.01
Self-Employed	486 (18.8%)	2114 (74.4%)		176 (16.7%)	915 (70.7%)		310 (20.2%)	1199 (77.5%)	
Employer	1579 (61.0%)	482 (17.0%)		605 (57.3%)	197 (15.2%)		974 (63.6%)	285 (18.4%)	
Employee	429 (16.6%)	70 (2.5%)		199 (18.8%)	32 (2.5%)		230 (15.0%)	38 (2.5%)	
Worker cooperative	32 (1.2%)	14 (0.5%)		18 (1.7%)	3 (0.2%)		14 (0.9%)	11 (0.7%)	
Domestic cleaning employee	58 (2.2%)	136 (4.8%)		54 (5.1%)	131 (10.1%)		4 (0.3%)	5 (0.3%)	
Unpaid family worker	4 (0.2%)	26 (0.9%)		4 (0.4%)	16 (1.2%)		0 (0.0%)	10 (0.6%)	
**Second Job**			0.40			0.69			0.59
No	2283 (88.2%)	2531 (89.1%)		943 (89.3%)	1167 (90.2%)		1340 (87.5%)	1364 (88.1%)	
Yes	298 (11.5%)	306 (10.8%)		107 (10.1%)	124 (9.6%)		191 (12.5%)	182 (11.8%)	
Missing	7 (0.3%)	5 (0.2%)		6 (0.6%)	3 (0.2%)		1 (0.1%)	2 (0.1%)	
**Number of employees in company**			<0.01			<0.01			<0.01
1 person	343 (13.3%)	1421 (50.0%)		176 (16.7%)	775 (59.9%)		167 (10.9%)	646 (41.7%)	
2-5 people	445 (17.2%)	900 (31.7%)		167 (15.8%)	346 (26.7%)		278 (18.1%)	554 (35.8%)	
6-20 people	415 (16.0%)	76 (2.7%)		242 (22.9%)	84.0 (6.5%)		368 (24.0%)	196 (12.7%)	
21-50 people	610 (23.6%)	280 (9.9%)		158 (15.0%)	25.0 (1.9%)		257 (16.8%)	51.0 (3.3%)	
>50 people	716 (27.7%)	87 (3.1%)		285 (27.0%)	27.0 (2.1%)		431 (28.1%)	60.0 (3.9%)	
Missing	59 (2.3%)	78 (2.7%)		28 (2.7%)	37 (2.9%)		31 (2.0%)	41 (2.6%)	
**In the last 4 weeks tried to change job**			<0.01			<0.01			<0.01
No	2359 (91.2%)	2390 (84.1%)		958 (90.7%)	1082 (83.6%)		1401 (91.4%)	1308 (84.5%)	
Yes	221 (8.5%)	443 (15.6%)		96.0 (9.1%)	206 (15.9%)		125 (8.2%)	237 (15.3%)	
Missing	8 (0.3%)	9 (0.3%)		2 (0.2%)	6 (0.5%)		6 (0.4%)	3 (0.2%)	
**Would like to work more hours**			<0.01			<0.01			<0.01
No	2118 (81.8%)	1931 (67.9%)		880 (83.3%)	886 (68.5%)		1238 (80.8%)	1045 (67.5%)	
Yes	452 (17.5%)	890 (31.3%)		168 (15.9%)	400 (30.9%)		284 (18.5%)	490 (31.7%)	
Missing	18 (0.7%)	21 (0.7%)		8 (0.8%)	8 (0.6%)		10 (0.7%)	13 (0.8%)	

a Informal employment defined as no contribution to social security systems. p values represent any group differences using t-tests (for mean [SD]), and chi-squared (for percents).

b Missing values: Overall (formal n = 11, informal = 38), Females (formal n = 3, informal n = 20), Males (formal n = 8, informal n = 18).

c The survey asked about the respondent's main job and the text responses were coded into occupation codes in accordance with the International Standard Classification of Occupations (ISCO-08). Three research team members reviewed the ISCO codes and combined them into broader categories through consensus: qualified nonmanual, nonqualified nonmanual, qualified manual, and nonqualified manual.

### 3.2 Multivariable models

*Overall.* In models adjusting for gender, age and education (model 1), individuals in informal jobs had a 29% higher prevalence of major depressive symptoms (Prevalence Ratio [PR]: 1.29; 95% Confidence Interval [CI]: 1.00, 1.65) compared to those in formal jobs (Table 3). This association was similar when controlling for household-level characteristics (PR: 1.27; 95% CI: 1.00, 1.62) (model 2).

**Table 3:** Association between informal employment and self-reported depressive symptoms.

		Prevalence Ratios (95% Confidence Interval)	
	N	Model 1^a^	Model 2^a^^,^^b^
Overall	5430	1.29 (1.00, 1.65)	1.27 (1.00, 1.62)
Female	2350	1.33 (1.03, 1.71)	1.36 (1.06, 1.74)
Male	3080	1.25 (0.92, 1.68)	1.22 (0.90, 1.65)

a Estimated using log-binomial models, employing a generalized estimating equation (GEE) approach, with city-clustered standard errors, adjusted for age and education; overall models also control for gender. Major depressive symptoms defined as CES-D-10 score $\underset{‾}{\text{>}}$ 16.

b Additionally adjusted for sociodemographic and household-level characteristics: relationship status, household size, having a child under the age of 5 in the household.

*Women.* The prevalence of major depressive symptoms among informally employed women was 33% higher compared to formally employed women, in our minimally adjusted model (PR: 1.33; 95% CI: 1.03, 1.71) and 36% higher in models adjusting for household-level characteristics (PR: 1.36; 95% CI: 1.06, 1.74).

*Men*. Although the association among men did not meet the traditional threshold for statistical significance, the magnitude of the association was substantively similar to that observed among women. Men in informal employment, compared to those formal employment, had a 25% higher prevalence of major depressive symptoms in our minimally adjusted model (PR: 1.25; 95% CI: 0.92, 1.68). Results were similar in magnitude when controlling for household-level characteristics (PR: 1.22; 95% CI: 0.90, 1.65).

In sensitivity analyses, using only respondents with a completed CES-D-10, the association between informal employment and major depressive symptoms was similar in direction, magnitude and statistical significance, as those observed in the analyses using imputed data (Supplemental Table 4). Results were also similar when excluding education as a covariate (Supplemental Table 5).

## 4 Discussion

We used a unique multi-city data source to test the association between informal employment and major depressive symptoms in 11 Latin American cities. Consistent with our hypothesis, those in informal employment compared to those in formal employment had a 27% higher prevalence of major depressive symptoms in our model adjusted for demographics and household-level characteristics. The magnitude of association between informal employment major depressive symptoms was substantively similar among women and men; however, the association only reached traditional thresholds of statistical significance among women.

Several prior studies suggest that informal employment is associated with poorer mental health (e.g., da Silva et al., 2006; Santana, 1997; Rodriguez-Loureiro et al., 2020). Similarly, we found that the association between informal employment and prevalence of depressive symptoms was significant overall. There are several plausible mechanisms through which informal employment may lead to poor mental health (Bolibar et al., 2021). Drawing on research on precarious employment and health from Europe (which has many shared characteristics as informal employment), prior studies suggest that material deprivation (i.e., insufficient wages) (Benach et al., 2007), perceived job insecurity (Ferrie et al., 2002; Witte, 1999) and the temporariness of employment (Bartoll et al., 2019; Virtanen et al., 2005) are associated with adverse mental health outcomes. Other pathways may include psychosocial stress, in part related to effort-reward-imbalance (e.g., high effort jobs with insufficient wages) (de Araújo et al., 2019; Siegrist & Marmot, 2004) that may exacerbate mental health disorders. Additionally, lower-quality employment could erode social support networks, which could act as a buffer to stressful events (Bolibar et al., 2021). Although these speculative mechanisms are plausible, we acknowledge that there are differences between LAC and European countries in terms of cultural norms, level of economic development, and social protections that may make the mechanistic framework for LAC slightly different from the Europe-derived mechanism.

Our study also found that the association between informal employment and major depressive symptoms were substantively similar in magnitude among women and men; while our study did not find a statistically significant association among men, we caution readers not to over-rely on p-values for interpretation of results (Amrhein et al., 2019) when the association was of similar magnitude. Nevertheless, prior studies reporting gender differences in the association between informal employment and mental health have yielded mixed results. Both Lopez-Ruiz et al. in Central America and Ludermir and Lewis in Brazil reported a positive association between informal employment and poorer mental health among women but not men (Lopez-Ruiz et al., 2017; Ludermir & Lewis, 2005). On the contrary, a study in Chile reported informal employment was associated with poorer mental health among men but not women (Ruiz et al., 2017). Mixed findings in the association by gender could partly be due to the different questionnaires used to measure mental health disorders (e.g., Self-Reporting Questionnaire (SRQ-20), 12-item General Health Questionnaire) or due to country-specific labor laws and welfare state models (e.g., type of governance system and social protection available) (Rodriguez-Loureiro et al., 2020; Utzet et al., 2021).

### 4.1 Policy implications

Our findings and those from previous studies support the need to address informal employment as a social determinant of health. Being employed in lower-quality jobs, with the limited legal and social protection, may exert more pressure on mental well-being. Governments have a role in developing economic and social policies to reduce the impact of working conditions in the informal sectors on physical and mental health as referenced directly the Sustainability Development Goal 8.3 “promote development-oriented policies that support productive activities, decent job creation, entrepreneurship, creativity and innovation, and encourage the formalization and growth of micro-, small- and medium-sized enterprises, including through access to financial services” United Nations. (n.d.) . Here, we briefly highlight a few from the literature for consideration.

First, governments should prioritize reducing informality by incorporating policies and strategies that balance economic growth and creation of high-quality jobs, transitioning informal jobs to formal employment (Ramirez, 2016). Several LAC countries have successfully implemented policies to drive economic growth, which helps to reduce informal work but at the same time instituted the needed legal and social protections for the informal workforce such as unemployment insurance, universal pension, healthcare insurance, and increasing the collective bargaining of trade unions and workers (Ramirez, 2016). Relating back the pathway by which precarious employment impacts mental health, increasing the availability of formal jobs with better wages, higher job security would help to decrease the prevalence of adverse mental health.

Second, policies may need to address and reduce the gender segmentation within informal workforce (e.g., few women in construction and transportation; more women in service orientated activities) and place of work (Chen et al., 2015) so that more women could participate in the workforce across various occupations and industries. Consistent with prior literature, our descriptive results show that more women were engaged in informal work than men (UN Women, 2015); this could be an indication that women might be bound by unpaid domestic work at home and/or other cultural norms, constraining their ability to find better employment opportunities. While both genders can benefit from policies creating more formal employment opportunities, such as higher education and job training, women could benefit from policies that reduce barriers for entry to the job market, such as social programs to address childcare needs and policies to ensure pay and benefit parity. Programs that provide economic benefits to employers that hire women or that meet a certain minimum proportion of women employed, including in leadership positions, could also help address women's informal employment disparities, while also benefiting those in formal employment (Kronfol et al., 2019). Policies addressing gender disparities in employment opportunities could help to improve mental health in LAC.

Third, promoting and supporting the formation of trade unions and associations among informal workers could increase their collective voices, visibility, and empowerment to influence government policies that meet their needs. For instance, Chen et al., 2015 reported that organized informal workers could enjoy benefits including negotiating for better wages and conditions, access to financial resources, accessing existing social protections, and increasing support systems. While challenges to organizing are equally prominent, successful policy campaigns from informal workers such as waste pickers in Bogotá, Colombia, or street vendors in India offer glimpse of hopes for informal workers (Chen et al., 2015). As discussed in the pathway, better wages and working conditions, and stronger social network through unions are conditions that help to decrease experiences of social insecurity and vulnerability that impact mental health.

Lastly, informal workers tend to be disproportionally burdened by mental health disorders but because of their low economic standing and limited or absent healthcare insurance, depending on the country, they might face more barriers accessing mental health resources and services compared to their formally employed counterparts. Guaranteeing a social safety net via universal health care or coverage, as well as ensuring there are enough mental health professionals available, could help informally employed individuals.

### 4.2 Study limitations

There are several limitations of this study. First, these data are cross-sectional, and thus, a causal relationship between informal employment and depressive symptoms cannot be established from our findings alone. Second, due to small sample sizes in some cities, we cannot include city fixed-effects and therefore, do not control for city- or country-level confounders (e.g., the pension systems). Third, the survey did not collect information on chronic illnesses, which could affect selection in certain occupations and depression risk and thus, we were not able to account for this plausible confounder in our models. Fourth, while our sample included 11 major cities in Latin America, it may not be generalizable to the entire working urban population in those cities. In addition, our results may be less generalizable to smaller Latin American cities and/or rural areas. Fifth, while the use of the social security/pension contribution as an indicator for informal employment is a commonly used indicator for informal employment in Latin American studies, it might not capture the multi-faceted aspects of the informal sector, and thus, it is possible that exposure misclassification could occur. However, this indicator has consistently been used in previous studies and by the ILO and we use the same indicator to help to facilitate comparison among studies and countries. Sixth, our sample had a considerable number of people who were inactive for more than three months and thus, were excluded. It is possible that some of these individuals were active informal workers who might work different jobs throughout the year because of the instability of informal work; in our study, the estimated prevalence of informal employment using contribution of social security/pension as the indicator is lower than informal employment by country in the literature. Finally, the CES-D-10 has been validated in few Latin American countries (e.g., indigenous populations in Boliva (Schantz et al., 2017) and Mexico (Franco-Díaz et al., 2018), but it is possible that cultural/contextual differences could affect the screening properties of the instrument. It should also be noted that the CES-D-10 is a screening instrument rather than diagnostic tool, thus, not all respondents with major depressive symptoms can be considered to have a diagnosis of clinical or major depression. Despite some limitations, investigating the association between informal employment and mental health across 11 Latin American cities allows for a broader picture of the relation across the region.

## 5 Conclusions

Given the widespread prevalence of informal employment in Latin American and the high prevalence of major depressive symptoms people in informal jobs face, it is important to advocate for policies to increase social protection for informal workers, whether via formalization of employment or the provision of universal social protection. Future research should investigate the association using longitudinal data to assess causality and delve into the mechanisms that might explain these associations, as well as the heterogeneity of results across genders.

## Declaration of conflicting of interests

The authors declared no potential conflicts of interest with respect to the research, authorship, and/or publication of this article.

## Financial disclosure

The Salud Urbana en América Latina (SALURBAL)/Urban Health in Latin America project is funded by the 10.13039/100010269Wellcome Trust [205177/Z/16/Z]. For the purpose of open access, the authors have applied a CC-BY public copyright license to any Author Accepted Manuscript version of this publication.

## Ethical statement

This study was approved by the Drexel University Institutional Review Board with ID #1612005035 and by appropriate site-specific IRBs across academic institutions included in the SALURBAL project.

## Authors contribution

**Tran B. Huynh** conceptualized, supervised, managed project, contributed to methodology, drafted the original draft. **Vanessa M. Oddo** conceptualized, contributed to methodology, and drafted the original draft. **Bricia Trejo** led the data analysis, visualization and supervised by **Kari Moore**. **Jannie Kim** contributed to the early data analysis**. D.** **Alex Quistberg** contributed to methodology, and data curation. **Francisco Diez-Canseco** contributed to methodology; **Alejandra Vives** contributed to conceptualization, methodology, and parts of the original draft. **All authors** critically reviewed, edited, and approved the final manuscript.
