# Supplementary material for: Association between informal employment and depressive symptoms in 11 cities in Latin America
Source: SSM Popul Health. Author manuscript; Available in PMC 2022 Jul 8. (PMC9187523; doi:10.1016/j.ssmph.2022.101101)
Supplement: Supplementary file [file EMS145883-supplement-Supplementary_file.docx]

**Supplemental Materials Tables**

**Association of informal employment and depressive symptoms in 11 urban cities in Latin America**

**Supplemental Table 1: Comparison of the characteristics of the final analytic sample to the total sample.**

**Supplemental Table 2: Overall selected sample characteristics by gender**

**Supplemental Table 3: Classification of Occupations**

**Supplemental Table 4: Association between informal employment and self-reported depressive symptoms using an unimputed sample**

**Supplemental Table 5: Association between informal employment and self-reported depressive symptoms excluding education as a covariate**

**Supplemental Table 1: Comparison of the characteristics of the final analytic sample to the total sample.**

|  | **Total Sample** | **Analytic Sample** | **Missing** | **chi-square  P-value^1^** |
| --- | --- | --- | --- | --- |
|  | **N=7979** | **N=5430** | **N=2549** |  |
| **Age** |  |  |  | 0.30 |
| 20-29 | 2123 (27%) | 1505 (28%) | 618 (24%) |  |
| 30-39 | 2399 (30%) | 1654 (30%) | 745 (29%) |  |
| 40-49 | 2016 (25%) | 1346 (25%) | 670 (26%) |  |
| 50-60 | 1436 (18%) | 925 (17%) | 511 (20%) |  |
| Missing | 5 (0%) | 0 (0%) | 5 (0%) |  |
| **Education** |  |  |  | 0.67 |
| Less than primary | 483 (6%) | 322 (6%) | 161 (6%) |  |
| Primary complete | 2401 (30%) | 1693 (31%) | 708 (28%) |  |
| Secondary complete | 3052 (38%) | 2048 (38%) | 1004 (39%) |  |
| Higher | 2014 (25%) | 1367 (25%) | 647 (25%) |  |
| Missing | 29 (0%) | 0 (0%) | 29 (1%) |  |
| **Gender** |  |  |  | 0.06 |
| Female | 3318 (42%) | 2350 (43%) | 968 (38%) |  |
| Male | 4657 (58%) | 3080 (57%) | 1577 (62%) |  |
| Missing | 4 (0%) | 0 (0%) | 4 (0%) |  |
| **Employment^2^** |  |  |  | 0.45 |
| Formal | 3749 (47%) | 2588 (48%) | 1161 (46%) |  |
| Informal | 4005 (50%) | 2842 (52%) | 1163 (46%) |  |
| Missing | 225 (3%) | 0 (0%) | 225 (9%) |  |

^1^ p values compare the analytic sample to total sample.

^2^ Informal employment defined as no contribution to social security systems.

**Supplemental Table 2: Overall selected sample characteristics by gender**

|  | **N (%) or Mean (Standard Deviation)^1^** | | |  |
| --- | --- | --- | --- | --- |
|  |  |  |  |  |
|  | **Female** | **Male** | **p-value** |  |
|  | **N=2,350** | **N=3,080** |  |  |
| **Employment type** |  |  | <0.01 |  |
| Formal | 1056 (44.9%) | 1532 (49.7%) |  |  |
| Informal^1^ | 1294 (55.1%) | 1548 (50.3%) |  |  |
| **Mean CES-D-10 Score^2^** | 8.9 (6.1) | 7.8 (5.7) | <0.01 |  |
| **Age** |  |  | 0.01 |  |
| 20-29 | 614 (26.1%) | 891 (28.9%) |  |  |
| 30-39 | 714 (30.4%) | 940 (30.5%) |  |  |
| 40-49 | 629 (26.8%) | 717 (23.3%) |  |  |
| 50-60 | 393 (16.7%) | 532 (17.3%) |  |  |
| **Education** |  |  | 0.02 |  |
| Less than Primary | 151 (6.4%) | 171 (5.6%) |  |  |
| Primary Complete | 695 (29.6%) | 998 (32.4%) |  |  |
| Secondary Complete | 873 (37.1%) | 1175 (38.1%) |  |  |
| > Secondary | 631 (26.9%) | 736 (23.9%) |  |  |
| **Mean Household size** | 4.1 (1.7) | 4.0 (1.8) | 0.27 |  |
| **Relationship Status** |  |  | <0.01 |  |
| In a relationship | 1564 (66.6%) | 2249 (73.0%) |  |  |
| Not in a relationship | 786 (33.4%) | 831 (27.0%) |  |  |
| **Children under 5 yrs present in household** |  |  | 0.28 |  |
| No | 1531 (65.1%) | 2051 (66.6%) |  |  |
| Yes | 819 (34.9%) | 1029 (33.4%) |  |  |
| **Mean Hours per week** | 41.7 (19.8) | 47.2 (16.7) | <0.01 |  |
| **Work Location** |  |  | <0.01 |  |
| At home | 514 (21.9%) | 314 (10.8%) |  |  |
| In a fixed kiosk in public street | 104 (4.4%) | 161 (5.2%) |  |  |
| No fixed place | 312 (13.3%) | 653 (21.2%) |  |  |
| Permanent place outside home | 1420 (60.4%) | 1932 (62.7%) |  |  |
| **Mean Years in Job** | 6.3 (7.5) | 8.2 (8.6) | <0.01 |  |
| **City** |  |  | <0.01 |  |
| Bogota | 380 (16.2%) | 339 (11.0%) |  |  |
| Buenos Aires | 291 (12.4%) | 423 (13.7%) |  |  |
| Caracas | 248 (10.6%) | 354 (11.5%) |  |  |
| Fortaleza | 208 (8.9%) | 238 (7.7%) |  |  |
| La Paz | 208 (8.9%) | 293 (9.5%) |  |  |
| Lima | 170 (7.2%) | 304 (9.9%) |  |  |
| Mexico | 110 (4.7%) | 189 (6.1%) |  |  |
| Montevideo | 252 (10.7%) | 263 (8.5%) |  |  |
| Panama | 73 (3.1%) | 117 (3.8%) |  |  |
| Quito | 176 (7.5%) | 252 (8.2%) |  |  |
| São Paulo | 234 (10.0%) | 308 (10.0%) |  |  |
| **Employment type** |  |  | <0.001 |  |
| Nonqualified manual | 812 (34.6%) | 873 (28.3%) |  |  |
| Nonqualified nonmanual | 1092 (46.5%) | 1007 (32.7%) |  |  |
| Qualified manual | 55 (2.3%) | 761 (24.7%) |  |  |
| Qualified nonmanual | 391 (16.6%) | 439 (14.3%) |  |  |

^1^ Informal employment was defined as no contribution to social security systems. P-values represent any group differences using t-tests (for mean [SD] and chi-squared (for percents).

^2^ Depression score: 10-item Center for Epidemiologic Studies Short Depression Scale

| **Supplemental Table 3: Classification of Occupations^1^** |
| --- |
| **Qualified Nonmanual** |
| 11 = Executive directors, management personnel of the public administration and member |
| 12 = Administrative and commercial directors |
| 13 = Directors and managers of production and operations |
| 14 = Managers of hotels, restaurants, shops and other services |
| 21 = Science and engineering professionals |
| 22 = Science and engineering professionals |
| 23 = Teaching professionals |
| 24 =Organization of public administration and companies specialists |
| 25 = Information technology and communications professionals |
| 26 = Professionals in law, social and cultural sciences |
| 31 = Mid-level science and engineering professionals |
| 32 = Mid-level health professionals |
| 33 = Mid-level professionals in financial and administrative operations |
| 34 = Mid-level professionals in legal, social, cultural and related services |
| 35 = Technicians of information and communications technology |
| **Nonqualified Nonmanual** |
| 41 = Clerks |
| 42 = Employees in direct contact with the public |
| 43 = Accountable employees and those in charge of registering materials |
| 44 = Other administrative support staff |
| 51 = Personal service workers |
| 52 = Sellers |
| 53 = Personal care workers |
| 54 = Personnel of protection services |
| **Qualified Manual** |
| 61 = Farmers and skilled workers |
| 62 = Qualified forest workers, fishermen and hunters |
| 71 = Officials and construction workers excluding electricians |
| 72 = Officers and operators of metallurgy, mechanical construction and related |
| 73 = Artisans and graphic artists |
| 74 = Workers specialized in electricity and electrotechnology |
| 83 = Vehicle drivers and operators of mobile heavy equipment |
| **Nonqualified Manual** |
| 63 = Farmers, fishermen, hunters and subsistence gatherers |
| 75 = Workers and officials of food processing, clothing, cabinetmakers |
| 81 = Operators of fixed installations and machines |
| 82 = Assemblers |
|  |
| 91 = Cleaners and assistants |
| 92 = Farming, fishing and forestry laborers |
| 93 = Mining, construction, manufacturing and transport laborers |
| 94 = Food preparation assistants |
| 95 = Street vendors of services and related |
| 96 = Waste collectors and other essential occupations |

^1^The survey asked about the respondent’s main job and the text responses were coded into occupation codes in accordance with the International Standard Classification of Occupations (ISCO-08). Three research team members reviewed the ISCO codes and combined them into broader categories through consensus: qualified nonmanual, nonqualified nonmanual, qualified manual, and nonqualified manual.

**Supplemental Table 4: Association between informal employment and self-reported depressive symptoms using an unimputed sample**

|  |  | **Prevalence Ratio (95% Confidence Interval)** | |
| --- | --- | --- | --- |
|  | **N** | **Model 1^1^** | **Model 2 ^1,2^** |
| Overall | 4,572 | 1.30 (0.93, 1.83) | 1.29 (0.92, 1.79) |
| Women | 1,985 | 1.42 (1.00, 2.03) | 1.46 (1.02, 2.07) |
| Men | 2,587 | 1.20 (0.83, 1.75) | 1.17 (0.80, 1.71) |

^1^ Estimated using log-binomial models, employing a generalized estimating equation (GEE) approach, with city-clustered standard errors, adjusted for sociodemographic characteristics: age and education; overall models also control for gender. Severe depressive symptoms defined as CES-D-10 score $\geq$ 16.

^2^ Additionally adjusted for sociodemographic and household-level characteristics: relationship status, household size, having a child under the age of 5 in the household.

**Supplemental Table 5: Association between informal employment and self-reported depressive symptoms, without education covariate**

|  |  | **Prevalence Ratios (95% Confidence Interval)** | |
| --- | --- | --- | --- |
|  | **N** | **Model 1^1^** | **Model 2^1,2^** |
| Overall | 5,430 | 1.35 (1.05, 1.73) | 1.33 (1.04, 1.70) |
| Female | 2,350 | 1.39 (1.05, 1.83) | 1.42 (1.10, 1.85) |
| Male | 3,080 | 1.31 (0.98, 1.74) | 1.27 (0.95, 1.71) |

^1^Estimated using log-binomial models, employing a generalized estimating equation (GEE) approach, with city-clustered standard errors, **adjusted for age**; overall models also control for gender. Major depressive symptoms defined as CES-D-10 score $\geq$ 16.

^2^Additionally adjusted for sociodemographic (age) and household-level characteristics: relationship status, household size, having a child under the age of 5 in the household.
